# Sickness absence trajectories following labour market participation patterns: a cohort study in Catalonia (Spain), 2012–2014

**DOI:** 10.1186/s12889-020-09396-9

**Published:** 2020-08-27

**Authors:** Julio C. Hernando-Rodriguez, Laura Serra, Fernando G. Benavides, Monica Ubalde-Lopez

**Affiliations:** 1 Center for Research in Occupational Health (CiSAL), Pompeu Fabra University, Barcelona, Spain; 2 CIBER of Epidemiology and Public Health (CIBERESP), Madrid, Spain; 3 IMIM – Parc Salut Mar, Barcelona, Spain; 4 GRECS-Research Group on Statistics, Econometrics and Health, Faculty of Economics and Business, University of Girona (UdG), C/ Universitat de Girona, 10, 17071 Girona, Spain; 5 Barcelona Institute for Global Health (ISGlobal), Barcelona, Spain

**Keywords:** Working life transition, Life course, Sick leave

## Abstract

**Background:**

Previous studies have focused on the relationship between employment pathways and health-related outcomes based on cross-sectional or longitudinal approaches. However, little is known about the cumulative effects of employment status mobility on sickness absence (SA) over time. The aim of the present study was to examine the association between prior labour market participation (LMP) patterns and SA trajectories from a life-course perspective.

**Methods:**

This cohort study was based on a sample of 11,968 salaried workers living in Catalonia and affiliated with the Spanish Social Security system, who accumulated more than 15 days on SA in at least one quarter during 2012–2014. Individuals were grouped into three different working life stages: early (18–25 years), middle (26–35 years), and late (36–45 years). To identify LMP patterns, we applied sequence analysis and cluster analysis (2002–2011), and we used latent class growth modelling to identify SA trajectories (2012–2014). Finally, we applied multinomial logistic regression models to assess the relationship between LMP patterns and SA trajectories.

**Results:**

The analyses yielded six LMP patterns: stable employment (value range: 63–81%), increasing employment (5–22%), without long-term coverage (7–8%), decreasing employment (4–10%), fluctuant employment (13–14%), and steeply decreasing employment (7–9%). We also identified four SA trajectories: low stable (83–88%), decreasing (5–9%), increasing (5–11%), and high stable (7–16%). However, the only significant association we identified for LMP patterns and SA trajectories was among young men, for whom an increasing employment pattern was significantly associated with a lower risk for increased days on SA (adjusted odds ratio: 0.21; 95% confidence interval: 0.05–0.96).

**Conclusions:**

SA trajectories are generally not related to prior 10-year LMP patterns at any stage of working life. To disentangle this relationship, future research might benefit from considering working life transitions with a quality-of-work approach framed with contextual factors closer to the SA course.

## Background

The ongoing phenomenon of increasing life expectancy and job insecurity affects the stability of the labour market structure [1]. Unstable employment pathways might entail the loss of economic resources, a lower likelihood of future employability when unemployed, and most important, loss of health. Experiences during working life are usually characterised as changes in employment and working conditions, the transitions in employment status that may influence an individual’s future health course. Such events and transitions may occur independently or be part of a cluster or causal chain, as each working experience increases the risk for other events or transitions. “Working life trajectory” references the co-occurrence of events through the life course, covering critical points in working life such as transitions in and out of the labour market and time spent in each state as a whole unit [2–4].

Previous research has mainly focused on risk and prognostic factors (individual, socio-demographic, occupational) for sickness absence (SA) [5–7], but a less considered aspect is the extent to which transitions in and out of the labour market may affect an individual’s work-related health outcomes. In the labour market context, a Norwegian study showed an inverse correlation between unemployment rate and the probability of having SA lasting longer than 14 days [8].

Together with socioeconomic, occupational, gender, and other individual factors, labour market transitions might differ with the stage of working life. For instance, young workers entering their working lives have greater mobility in terms of more gaps and a larger number of transitions between different employment statuses (i.e., employment, unemployment, or inactivity spells) compared with those in a late career stage [9]. In keeping with this pattern, a study of employment patterns among women in Germany showed that younger cohorts followed a trend towards discontinuous and part-time careers, whereas careers involving continuous full-time employment or being a housewife were becoming unusual [10].

Traditionally, for health and health-related outcomes, occupational epidemiology has assessed exposure–outcome association measures, whether simple or accumulated, at one point in time. Few studies have involved longitudinal analyses of the effect of prior working life on health [11–16]. From the standpoint of occupational epidemiology, because a person lives in a multidimensional and multilevel context, an approach from a life-course perspective could elucidate relevant events in a person’s working life that manifest and shape health status over time [17]. The extent to which prior labour market participation (LMP) patterns affect risk for future SA remains unexplored [2, 4].

Previous studies have examined sex and age differences in health and health-related outcomes and in LMP [18–21]. The authors of one review found that women have higher short-term SA rates than men, with variations by country and age cohort. Likewise, higher proportions of SA affected the beginning and middle parts of working life among women ages 20–54 years compared to men of the same ages, which could be related to pregnancy-related health problems or psychological distress when employed in occupations where women are underrepresented [19]. Because cohorts reflect exposures to past socioeconomic circumstances and institutional contexts, considering several cohorts may uncover different influences across a life course [22]. For this reason, a separate analysis by sex and by cohort, defined by working life stage, could contribute to disentangling the relationship between LMP and SA over a lifetime.

We hypothesised that prior highly fragmented LMP patterns would be associated with more unfavourable later SA trajectories compared to LMP patterns suggesting more stable employment pathways. We also hypothesised that this relationship might differ across working life stages and by sex. In this analysis, we viewed fragmented LMP patterns as being characterised by multiple transitions from employment to unemployment, either with benefits or without Social Security coverage.

The aim of this study was to investigate the relationship between prior LMP patterns at the early, middle, and late stages of working life and subsequent SA trajectories among individuals who had accumulated more than 15 days of SA in at least one of the quarters during 2012–2014, considering occupational and socioeconomic characteristics and SA medical diagnosis categories as potential confounders.

## Methods

### Study population

The study population was part of the Spanish WORKing life Social Security cohort (WORKss cohort), an annual random representative sample of 4% of affiliates with the Spanish Social Security system. The sampling has taken place at least one day a year starting in 2004, and the information includes employment history register data from 1981 [23]. The sample is updated annually following an algorithm, which selects the same individuals if they continue affiliated with the Spanish Social Security system. Those lost because of administrative inactivity or death are replaced with members from the target population until the sample again reaches 4%, which ensures sample representativeness. We obtained data related to working life from the WORKss cohort information and SA records for 2012–2014 from the Catalan Institute of Medical Evaluations, which contains data only for people residing in Catalonia. SA records include information on the starting/closure date of SA episodes and medically certified diagnoses coded using the International Classification of Diseases 10th version (ICD-10).

This study was based on a cohort of 11,968 salaried workers living in Catalonia and affiliated with the Spanish Social Security system, who accumulated more than 15 days on SA in at least one quarter during 2012–2014. Their prior working lives from 2002 to 2011 were reconstructed, and they were grouped into three working life stages according to their age in 2002: early working life cohort (WLC; 18–25 years, 33.3%); middle WLC (26–35 years, 37.5%), and late WLC (36–45 years, 29.2%). Approximately 75% of the SA episodes included in these data were shorter than 15 days, mainly represented by acute diagnoses (e.g., infectious and respiratory diseases). The rationale for selecting only workers who accumulated more than 15 days on SA is that SA episodes of this length are more likely to represent severe rather than mild or moderate diagnoses (half of SA episodes lasting longer than 15 days are the result mainly of mental and musculoskeletal disorders) [24]. For this reason, severe SA episodes may better reflect the potentially long-lasting effects of prior LMP patterns. This duration of episode also allows for exclusion of an influence from SA monetary benefits on a person’s SA behaviour because long-term SA represents serious illness [25].

### Variables

LMP patterns were defined based on weekly transitions among six labour market states: *employment*, *unemployment insurance benefits*, *means-tested unemployment benefits*, *transition*, *without coverage*, and *without long-term coverage*. The Spanish unemployment benefit scheme distinguishes two categories of unemployment, unemployment receiving benefits and means-tested unemployment benefits. Entitlement to unemployment insurance benefits requires previously paid contributions into Social Security of at least one year over the last 6 years. The duration depends on the paid contributions, and the amount is based on the average wage before becoming unemployed and the replacement rate (70% in the first 6 months, 50% afterwards). Means-tested unemployment benefits can be claimed after unemployment insurance benefits are exhausted or when individuals do not fulfil the conditions for receiving entitled benefits. The duration depends on the time contributed, and the amount is lower than the minimum wage [26, 27]. We maintained the separation of the two unemployment categories, with means-tested benefits as the least generous, which may allow for capture in the LMP patterns of situations involving more vulnerable workers. For situations of employment and unemployment receiving unemployment benefits/means-tested unemployment benefits, individuals keep contact with the Social Security system. Periods without records in the Social Security registry were categorised ad hoc into three states. The state *transition* was defined as a period between employment states up 30 days (i.e., administrative transition) [16]. The state *without coverage* refers to periods between employment states longer than 30 days. The state *without long-term coverage* accounts for the first labour market entry or return to work (i.e., left censorship) and/or a labour market withdrawal or a temporary leaving (i.e., right censorship). SA trajectories were based on the sum of days on SA each quarter between 2012 and 2014 of workers who had accumulated more than 15 days on SA any quarter.

Included covariates for the period 2012–2014 were type of contract (permanent and temporary), working time (full-time, part-time 51–99%, and part-time up to 50%), occupational category (skilled non-manual, skilled manual, unskilled non-manual, and unskilled manual), income (average monthly income categorised into quartiles), and SA medical diagnosis (grouped according to the ICD-10). The diagnosis groups included were mental disorders (F00-F99), digestive diseases (K00-K93), musculoskeletal diseases (M00-M99), pregnancy (O00-O99), injuries and poisoning (S00-T98), and others (the remaining codes). Diagnosis groups that included few people or none in the SA trajectories, such as acute (i.e., infections, circulatory diseases, respiratory diseases) or unusual according to their nature (i.e., pregnancies in women from the late WLC and neoplasms) were excluded.

Because workers could transit among different employment conditions and diagnosis groups during the SA trajectory during 2012 to 2014, we assigned them to the category where they spent most of the time for the whole follow-up period. Similarly, because workers had daily labour market states during the prior LMP, 2002–2011, we assigned them to the state where they spent most of the time for each week of the period.

Income was based on the monthly salary and unemployment benefits, subject to legal limits. The average monthly income was the total remuneration a worker received divided by time employed monthly. Additionally, time employed measured in the years 2002–2011 was used as an adjustment variable.

### Statistical analyses

All the analyses were performed for each sex and WLC group separately. In a first step, we reconstructed the prior 10 years of LMP patterns by applying sequence analysis based on the six previously defined states. This methodology allowed us to describe individual sets of state sequences [28]. An optimal matching approach supported development of a matrix of distances between each sequence given indels (i.e., insertion and deletion) together with substitution costs. A custom substitution costs matrix was defined according to the characteristics of our data and study population, with a higher weight given to the transitions that were considered less frequent (i.e., from employment to means-tested unemployment benefits, or from employment to without coverage, and vice versa). Then, we used hierarchical cluster analysis to group similar sequences (i.e., LMP patterns) [29]. We based our selection of the optimal number of clusters on the average silhouette width (ASW), which allows for the assessment of clustering validity (ASW > 0.5 indicates reasonably well-separated clusters) (Supplementary Table 1) [30, 31].

In a second step, we identified SA trajectories using latent class growth analysis (LCGA) based on accumulating more than 15 days on SA quarterly (if any) during the 3-year follow-up, specifying a linear functional form. An assumption of LCGA is that individuals within a trajectory are homogeneous, and individuals are assigned into subgroups with similar characteristics, according to a membership probability [32]. The optimal number of trajectories was assessed considering the Bayesian information criterion (lower best fit) and the Lo-Mendell-Rubin adjusted likelihood ratio test and bootstrap likelihood ratio test. A minimum of 1% of the total sample is recommended for each class [33], although this rule depends on the sample size and how meaningful a small group is for the study aim [34]. Additionally, high posterior probabilities (> 0.7) and researcher criteria were applied to determine the final number of trajectories. In cases of similar values for compared goodness-of-fit indices, we selected the one with the highest entropy (near 1.0) (Supplementary Table 2) [34]. We also assessed model adequacy indices using odds of correct classification (> 5 for all groups indicates high assignment accuracy) and mismatch scores (values close to 0 indicate individuals were assigned to groups with high certainty) (Supplementary Table 3) [35].

In a final step, we examined the association between LMP patterns and SA trajectories, using multinomial logistic regression models adjusted for potential confounders. We applied TraMineR package in R for the Sequence Analysis, MPlus v.7 for the LCGA, and Stata v.13© for the multinomial logistic regression analysis.

## Results

**Fig. 1:**
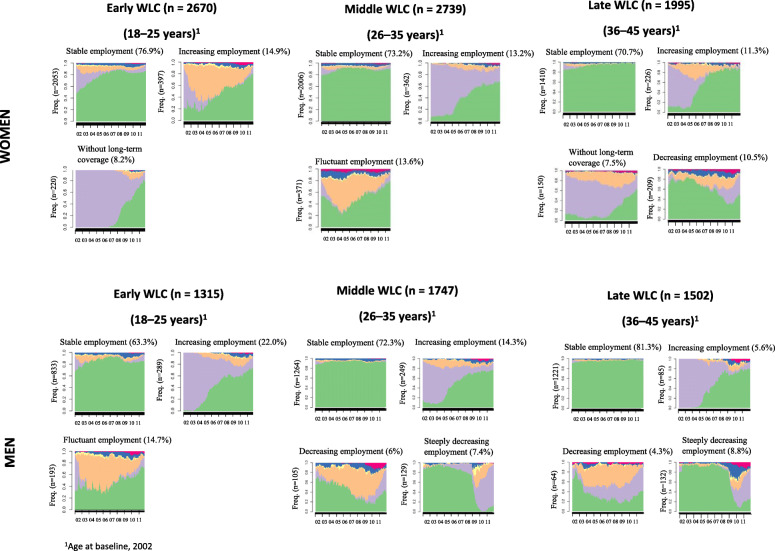
Sickness absence trajectories (> 15 accumulated days on sickness absence per quarter) in salaried workers across working life cohorts (WLCs) (N = 11,968). Catalonia, 2012–2014

The analysis identified six LMP patterns (Fig. 1): stable employment (value range: 63.3–81.3% of workers), increasing employment (5.6–22%), without long-term coverage (7.5–8.2%), decreasing employment (4.3–10.5%), fluctuant employment (13.6–14.7%), and steeply decreasing employment (7.4–8.8%), with 3–4 patterns in each sex and WLC group. The stable employment LMP pattern showed a higher proportion of women in the early WLC compared to proportions in the late WLC (76.9% vs 70.7%), whereas the opposite pattern was observed for men (63.3% vs 81.3%). We also identified other different LMP patterns by sex, including a pattern without long-term Social Security coverage in women only, and a steeply decreasing employment pattern only among men.

**Fig. 2:**
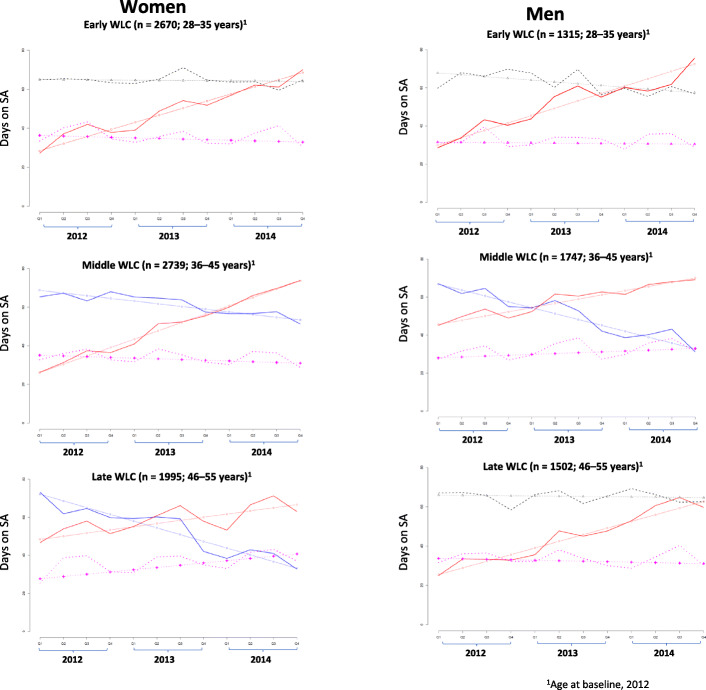
Labour market participation patterns in salaried workers with future sickness absence (> 15 accumulated days on sickness absence per quarter) across working life cohorts (WLCs) (*N* = 11,968). Catalonia, 2002–2011. Women – Early WLC . Women – Middle WLC . Women – Late WLC . Men – Early WLC . Men – Middle WLC . Men – Late WLC

Regarding the SA trajectories, we identified four (Fig. 2): low stable (82.9–88.1% of individuals), decreasing (5–9.4%), increasing (0.8–11.3%), and high stable (6.7–16.3%), with three trajectories in each sex and WLC group. The mean of accumulated days on SA ranged from 30 to 40 days in the low stable trajectory to 60 to 70 days in the high stable trajectory.

Across SA trajectories, we also found significant differences by type of contract and diagnosis group. Among women, compared to other groups, a higher proportion had temporary contracts in the high stable SA trajectory in the early WLC (28.5%) and the increasing SA trajectory in the middle WLC (26%). The increasing trajectory had the highest proportion of women who accumulated days on SA because of mental disorders in the early and middle WLCs (24.5 and 28%, respectively). The decreasing and increasing SA trajectories showed a similar proportion of workers on SA because of mental disorders among women in the late WLC (20 and 21.1%, respectively) and men in the middle WLC (15.9 and 16.5%, respectively).

Otherwise, the distribution of LMP patterns was mainly homogeneous across SA trajectories. Among women in the middle WLC and men in the early WLC, the increasing SA trajectories showed a higher proportion of workers who had a prior stable employment LMP pattern (78.1 and 71.4%, respectively) compared to other trajectories. For men in the middle WLC, the decreasing SA trajectory showed a lower proportion of individuals with the LMP pattern of increasing employment (8.4%) compared to the low stable (14.7%) and increasing trajectories (14.1%) (Tables 1 and 2).

**Table 1:** Distribution of salaried women from early, middle, and late working life cohorts (WLCs) (*N* = 7404) across sickness absence trajectories (> 15 accumulated days on sickness absence per quarter) by employment conditions, occupational category, diagnosis groups, and the prior 10-year labour market participation patterns. Catalonia, 2012–2014

	Early WLC (***N*** = 2670)				Middle WLC (***N*** = 2739)				Late WLC (***N*** = 1995)			
**WOMEN**	**Low stable (87.4%)**	**High stable (6.7%)**	**Increasing (5.9%)**	*p*^a^	**Low stable (85.3%)**	**Decreasing (9.4%)**	**Increasing (5.3%)**	*p*^a^	**Low stable (85.4%)**	**Decreasing (5.0%)**	**Increasing (9.6%)**	*p*^a^
	N (%)	N (%)	N (%)		N (%)	N (%)	N (%)		N (%)	N (%)	N (%)	
**Type of contract**												
Permanent contract	1852 (79.4)	128 (71.5)	125 (79.1)	.045	1935 (82.8)	207 (80.5)	108 (74.0)	.020	1484 (87.0)	85 (85.9)	168 (88.0)	.876
Temporary contract	481 (20.6)	51 (28.5)	33 (20.9)		401 (17.2)	50 (19.5)	38 (26.0)		221 (13.0)	14 (14.1)	23 (12.0)	
**Working time**												
Full-time	1526 (65.4)	114 (63.7)	101 (63.9)	.984	1528 (65.4)	160 (62.2)	98 (67.1)	.787	1274 (74.7)	72 (72.8)	145 (76.0)	.771
Part-time: > 50% up to 99%	602 (25.8)	49 (27.4)	43 (27.2)		568 (24.3)	66 (25.7)	32 (21.9)		251 (14.7)	14 (14.1)	23 (12.0)	
≤ 50%	205 (8.8)	16 (8.9)	14 (8.9)		240 (10.3)	31 (12.1)	16 (11.0)		180 (10.6)	13 (13.1)	23 (12.0)	
**Occupational category**												
Skilled non-manual	562 (24.5)	42 (23.8)	31 (20.0)	.305	521 (22.7)	58 (23.3)	42 (28.7)	.556	291 (17.5)	15 (15.5)	28 (15.1)	.759
Skilled manual	327 (14.2)	34 (19.2)	25 (16.1)		351 (15.3)	36 (14.5)	23 (15.8)		285 (17.2)	18 (18.6)	31 (16.8)	
Unskilled non-manual	1248 (54.2)	85 (48.0)	90 (58.1)		1172 (51.1)	127 (51.0)	62 (42.5)		765 (46.0)	41 (42.2)	94 (50.8)	
Unskilled manual	164 (7.1)	16 (9.0)	9 (5.8)		250 (10.9)	28 (11.2)	19 (13.0)		321 (19.3)	23 (23.7)	32 (17.3)	
**Income in quartiles**												
High	794 (34.1)	50 (27.9)	54 (34.1)	.229	733 (31.3)	69 (27.0)	40 (27.4)	.152	475 (27.9)	26 (26.2)	43 (22.5)	.625
Middle-high	721 (30.9)	51 (28.6)	50 (31.7)		705 (30.2)	78 (30.4)	55 (37.7)		555 (32.6)	28 (28.3)	67 (35.1)	
Middle-low	532 (22.8)	57 (31.8)	36 (22.8)		569 (24.4)	69 (27.0)	39 (26.7)		423 (24.8)	28 (28.3)	54 (28.3)	
Low	284 (12.2)	21 (11.7)	18 (11.4)		328 (14.1)	40 (15.6)	12 (8.2)		251 (14.7)	17 (17.2)	27 (14.1)	
**Diagnosis group (ICD-10)**												
Mental	252 (11.8)	32 (18.8)	36 (24.5)	<.001	294 (14.9)	52 (22.3)	35 (28.0)	.001	235 (16.8)	15 (20.0)	36 (21.1)	.008
Digestive	83 (3.9)	5 (2.9)	3 (2.0)		131 (6.6)	12 (5.2)	3 (2.4)		99 (7.0)	3 (4.0)	10 (5.8)	
Musculoskeletal	925 (43.3)	44 (25.7)	47 (32.0)		638 (32.4)	68 (29.2)	40 (32.0)		459 (32.6)	28 (37.3)	65 (38.0)	
Pregnancy	327 (15.3)	39 (22.8)	30 (20.4)		195 (9.9)	28 (12.0)	13 (10.4)		–	–	–	
Injuries and poisoning	173 (8.1)	14 (8.2)	8 (5.4)		209 (10.6)	27 (11.6)	13 (10.4)		211 (15.0)	17 (22.7)	32 (18.7)	
Others	377 (17.6)	37 (21.6)	23 (15.7)		505 (25.6)	46 (19.7)	21 (16.8)		403 (28.6)	12 (16.0)	28 (16.4)	
**Labour market participation patterns**												
Stable employment	1789 (76.7)	143 (79.9)	121 (76.6)	.814	1706 (73.0)	186 (72.4)	114 (78.1)	.701	1214 (71.2)	66 (66.7)	130 (68.1)	.728
Increasing employment	347 (14.9)	25 (14.0)	25 (15.8)		310 (13.3)	37 (14.4)	15 (10.3)		186 (10.9)	12 (12.1)	28 (14.7)	
Without long-term coverage (early and late)/Fluctuant employment (middle)	197 (8.4)	11 (6.2)	12 (7.6)		320 (13.7)	34 (13.2)	17 (11.6)		127 (7.5)	8 (8.1)	15 (7.9)	
N/A (early and middle)/Decreasing employment (late)	N/A	N/A	N/A		N/A	N/A	N/A		178 (10.4)	13 (13.1)	18 (9.4)	
Total	2333 (100)	179 (100)	158 (100)		2336 (100)	257 (100)	146 (100)		1705 (100)	99 (100)	191 (100)	

Missing values in the occupational category (OC), income (I), and diagnosis group (DG): N (%) in the early cohort (low stable—OC: 32 (1.37); I: 2 (0.09); DG: 1 (0.04); increasing—OC: 3 (1.90); high stable—OC: 2 (1.12)); middle cohort (low stable—OC: 42 (1.80); I: 1 (0.04); DG: 5 (0.21); decreasing—OC: 8 (3.11); I: 1 (0.39)); and late cohort (low stable—OC: 43 (2.52); I: 1 (0.06); DG: 1 (0.06); decreasing—OC: 2 (2.02); increasing—OC: 6 (3.14)) by sickness absence trajectory in the period 2012–2014. Income in quartiles based on the average monthly income in the early cohort (high: 5063€; middle-high: 1940€; middle-low: 1337€; low: 886€), middle cohort (high: 5315€; middle-high: 2127€; middle-low: 1403€; low: 893€), and late cohort (high: 5577€; middle-high: 2151€; middle-low: 1386€; low: 856€). ^a^Chi-squared tests. ^b^Fisher’s exact tests.

**Table 2:** Distribution of salaried men from early, middle, and late working life cohorts (WLCs; *N* = 4564) across sickness absence trajectories (> 15 accumulated days on sickness absence per quarter) by employment conditions, occupational category, diagnosis groups, and the prior 10-year labour market participation patterns in Catalonia, 2012–2014

MEN	Early WLC (***N*** = 1315)				Middle WLC (***N*** = 1747)				Late WLC (***N*** = 1502)			
	Low stable (88.1%)	High stable (8.7%)	Increasing (3.2%)	*p*^a^	Low stable (83.3%)	Decreasing (5.4%)	Increasing (11.3%)	*p*^a^	Low stable (82.9%)	High stable (16.3%)	Increasing (0.8%)	*p*^a^
	N (%)	N (%)	N (%)		N (%)	N (%)	N (%)		N (%)	N (%)	N (%)	
**Type of contract**												
Permanent contract	923 (79.6)	90 (78.9)	31 (73.8)	.652	1228 (84.5)	81 (85.3)	162 (81.8)	.607	1114 (89.5)	212 (86.5)	12 (100.0)	.191
Temporary contract	236 (20.4)	24 (21.1)	11 (26.2)		226 (15.5)	14 (14.7)	36 (18.2)		131 (10.5)	33 (13.5)	0	
**Working time**												
Full-time	1018 (87.8)	101 (88.6)	35 (83.3)	.551^b^	1311 (90.1)	84 (88.4)	178 (89.9)	.245	1167 (93.7)	233 (95.1)	10 (83.4)	.129^b^
Part-time: > 50% up to 99%	66 (5.7)	7 (6.1)	5 (11.9)		72 (5.0)	4 (4.2)	5 (2.5)		33 (2.7)	8 (3.3)	1 (8.3)	
≤ 50%	75 (6.5)	6 (5.3)	2 (4.8)		71 (4.9)	7 (7.4)	15 (7.6)		45 (3.6)	4 (1.6)	1 (8.3)	
**Occupational category**												
Skilled non-manual	137 (11.8)	7 (6.1)	2 (4.8)	.167	232 (16.0)	14 (14.7)	21 (10.6)	.224	252 (20.3)	46 (18.9)	1 (8.4)	.161^b^
Skilled manual	514 (44.4)	46 (40.4)	22 (52.3)		599 (41.1)	40 (42.1)	91 (46.0)		502 (40.3)	94 (38.4)	3 (25.0)	
Unskilled non-manual	348 (30.0)	41 (36.0)	10 (23.8)		440 (30.3)	26 (27.4)	68 (34.3)		382 (30.7)	76 (31.2)	4 (33.3)	
Unskilled manual	160 (13.8)	20 (17.5)	8 (19.1)		183 (12.6)	15 (15.8)	18 (9.1)		108 (8.7)	28 (11.5)	4 (33.3)	
**Income in quartiles**												
Highest	347 (29.9)	28 (24.6)	9 (21.4)	.131	427 (29.3)	24 (25.3)	48 (24.2)	.260	349 (28.1)	55 (22.4)	4 (33.3)	.314^b^
Third	359 (31.0)	32 (28.1)	11 (26.2)		466 (32.1)	34 (35.8)	56 (28.3)		400 (32.1)	96 (39.2)	5 (41.7)	
Second	290 (25.0)	34 (29.8)	10 (23.8)		344 (23.7)	25 (26.3)	55 (27.8)		324 (26.0)	58 (23.7)	2 (16.7)	
Lowest	163 (14.1)	20 (17.5)	12 (28.6)		217 (14.9)	12 (12.6)	39 (19.7)		172 (13.8)	36 (14.7)	1 (8.3)	
**Diagnosis group (ICD-10)**												
Mental	151 (14.8)	17 (15.9)	10 (23.8)	.467	146 (11.9)	14 (15.9)	29 (16.5)	.001	107 (10.4)	26 (12.3)	1 (9.1)	.051^b^
Digestive	94 (9.2)	7 (6.6)	4 (9.5)		188 (15.3)	3 (3.4)	9 (5.1)		184 (17.9)	19 (9.1)	2 (18.1)	
Musculoskeletal	264 (25.9)	27 (25.2)	12 (28.6)		316 (25.7)	21 (23.9)	50 (28.4)		313 (30.5)	76 (36.4)	3 (27.3)	
Injuries and poisoning	297 (29.2)	38 (35.5)	12 (28.6)		333 (27.1)	30 (34.1)	59 (33.5)		149 (14.5)	35 (16.8)	3 (27.3)	
Others	213 (20.9)	18 (16.8)	4 (9.5)		245 (20.0)	20 (22.7)	29 (16.5)		274 (26.7)	53 (25.4)	2 (18.2)	
**Labour market articipation patterns**												
Stable employment	735 (63.4)	68 (59.7)	30 (71.4)	.309	1045 (71.9)	72 (75.8)	147 (74.2)	.587	1013 (81.4)	197 (80.4)	11 (91.7)	.923^b^
Increasing employment	258 (22.3)	27 (23.7)	4 (9.5)		213 (14.7)	8 (8.4)	28 (14.1)		69 (5.5)	16 (6.5)	0	
Fluctuant employment (early)/decreasing employment (middle and late)	166 (14.3)	19 (16.7)	8 (19.1)		90 (6.2)	5 (5.3)	10 (5.1)		56 (4.5)	8 (3.3)	0	
N/A (early)/Steeply decreasing employment (middle and late)	N/A	N/A	N/A		106 (7.3)	10 (10.5)	13 (6.6)		107 (8.6)	24 (9.8)	1 (8.3)	
Total	1159 (100)	114 (100)	42 (100)		1454 (100)	95 (100)	198 (100)		1245 (100)	245 (100)	12 (100)	

Missing values in the occupational category (OC), income (I), and diagnosis group (DG): N (%) in the early cohort (low stable—DG: 1 (0.09); high stable—DG: 2 (1.75)); middle cohort (low stable—DG: 3 (0.21)); and late cohort (low stable—OC: 1 (0.08); DG: 2 (0.16); high stable—OC: 1 (0.41)) by sickness absence trajectory in the period 2012–2014. Income in quartiles based on the average monthly income in the early cohort (high: 5247€; middle-high: 2205€; middle-low: 1560€; low: 1076€), middle cohort (high: 6577€; middle-high: 2724€; middle-low: 1845€; low: 1264€), and late cohort (high: 6339€; middle-high: 3094€; middle-low: 2027€; low: 1419€). ^a^Chi-squared tests. ^b^Fisher’s exact tests.

Adjusted regression models did not show significant associations between the prior 10 years of LMP patterns and subsequent SA trajectories (Table 3). Only men in the early WLC who had an increasing employment LMP pattern showed a significantly lower risk for increased accumulated days on future SA compared to those who had continuous stable employment LMP patterns (adjusted odds ratio: 0.21; 95% confidence interval: 0.04–0.96).

**Table 3:** Association between labour market participation patterns and sickness absence trajectories (> 15 accumulated days on sickness absence per quarter) in salaried workers from early, middle, and late working life cohorts (WLCs; N = 11,968). Catalonia (Spain), 2012–2014 (odds ratios [ORs] and 95% confidence intervals [CIs])

WOMEN			Sickness absence trajectories^**a**^			
	Early WLC (N = 2670)		Middle WLC (N = 2739)		Late WLC (N = 1995)	
	High stable vs low stable	Increasing vs low stable	Decreasing vs low stable	Increasing vs low stable	Decreasing vs low stable	Increasing vs low stable
	OR (95% CI)	OR (95% CI)	OR (95% CI)	OR (95% CI)	OR (95% CI)	OR (95% CI)
**Labour market participation patterns**						
**Crude model**						
Stable employment	1	1	1	1	1	1
Increasing employment	0.90 (0.58–1.40)	1.07 (0.68–1.66)	1.09 (0.75–1.59)	0.72 (0.42–1.26)	1.19 (0.63–2.24)	1.41 (0.91–2.18)
Without long-term coverage (early and late) /Fluctuant employment (middle)	0.70 (0.37–1.31)	0.90 (0.49–1.66)	0.97 (0.66–1.43)	0.80 (0.47–1.34)	1.16 (0.54–2.47)	1.10 (0.63–1.94)
N/A (early and middle)/Decreasing employment (late)	N/A	N/A	N/A	N/A	1.34 (0.73–2.49)	0.94 (0.56–1.58)
**Adjusted model** ^**b**^						
Stable employment	1	1	1	1	1	1
Increasing employment	0.80 (0.46–1.38)	1.35 (0.77–2.37)	1.07 (0.57–2.01)	1.10 (0.47–2.60)	1.21 (0.37–3.96)	0.92 (0.42–2.02)
Without long-term coverage (early and late) /Fluctuant employment (middle)	0.56 (0.24–1.32)	1.13 (0.46–2.78)	0.89 (0.51–1.55)	1.13 (0.55–2.32)	1.40 (0.21–9.13)	0.56 (0.15–2.06)
N/A (early and middle)/Decreasing employment (late)	N/A	N/A	N/A	N/A	0.90 (0.31–2.59)	0.70 (0.34–1.45)
**MEN**	**Early WLC (N = 1315)**		**Middle WLC (N = 1747)**		**Late WLC (N = 1502)**	
	**High stable vs low stable**	**Increasing vs low stable**	**Decreasing vs low stable**	**Increasing vs low stable**	**High stable vs low stable**	**Increasing vs low stable**
**Crude model**						
Stable employment	1	1	1	1	1	1
Increasing employment	1.13 (0.71–1.81)	0.38 (0.13–1.09)	0.55 (0.26–1.15)	0.93 (0.61–1.44)	1.19 (0.68–2.10)	–
Fluctuant employment (early)/Decreasing employment (middle and late)	1.24 (0.72–2.11)	1.18 (0.53–2.62)	0.81 (0.32–2.05)	0.79 (0.40–1.55)	0.73 (0.34–1.57)	–
N/A (early)/Steeply decreasing employment (middle and late)	N/A	N/A	1.37 (0.69–2.73)	0.87 (0.48–1.59)	1.15 (0.72–1.84)	0.86 (0.11–6.73)
**Adjusted model** ^**b**^						
Stable employment	1	1	1	1	1	1
Increasing employment	1.27 (0.58–2.77)	0.21 (0.05–0.96)	0.79 (0.26–2.44)	0.97 (0.46–2.04)	1.73 (0.59–5.03)	–
Fluctuant employment (early) /Decreasing employment (middle and late)	1.20 (0.56–2.59)	0.74 (0.23–2.34)	1.11 (0.30–4.10)	0.78 (0.30–2.01)	1.42 (0.42–4.80)	–
N/A (early)/ Steeply decreasing employment (middle and late)	N/A	N/A	1.81 (0.73–4.47)	0.94 (0.45–1.96)	1.38 (0.74–2.59)	0.76 (0.06–0.34)

The low stable class is the reference group for the multinomial logistic regression analysis; ^b^model adjusted for type of contract, working time, occupational category, income in quartiles, diagnosis group during 2012–2014, and time of employment during 2002–2011.

## Discussion

Our main finding was that the four SA trajectories (low stable, decreasing, increasing, and high stable) were not related to the six prior 10-year LMP patterns across the three stages of working life considered. More than 80% of workers showed a low stable accumulation of 30 to 40 days on SA on average during the 3-year follow-up.

The result was unexpected, according to our hypothesis, because it is reasonable to consider that the accumulation of adverse employment status (frequent entries into and exits from the labour market) during a previous working life could be related to an increase in SA days because of more severe or long-lasting pathologies (i.e., chronic diseases such as musculoskeletal and mental disorders). However, the result persists after adjustment for socioeconomic and employment-related factors and medical diagnosis during the SA course, with this last emerging as a leading determinant of the SA trajectory [36].

As far as we know, no previous studies have examined the relationship between prior 10-year LMP patterns and future SA trajectories from a life-course perspective. A Finnish study focused on early exits from employment, identifying and describing 10-year working life participation patterns and determining the cumulative incidence of SA within these patterns. Those authors found that individuals with long-term labour market exit had the highest cumulative incidence of SA because of mental disorders [20]. Similarly, one investigation of the effect of precarious employment on SA in four Nordic countries showed that precarious employment was associated with SA of 7 days or more, and another report on health-related outcomes (including long-term SA) in 28 countries in Europe cited an association with SA of more than 15 days [37, 38]. In both studies, however, the authors measured precarious employment as a multidimensional construct based on indicators obtained from several dimensions (e.g., employment instability, lack of power and rights, reduced salary), which captured the accumulation of unfavourable aspects of employment quality [39]. Therefore, the results derived from these studies may not be directly comparable to the current findings from our analyses of LMP patterns based on employment status mobility across different stages of the working life course.

Previous groups also have analysed the possible association between certain labour market situations and several health-related outcomes. One group found in a follow-up of 15 years that unemployment at an early age had a dose-response relationship with increased risk of SA, disability pension, and death [40]. Another study showed that a high number of periods without Social Security coverage was the main predictor of early retirement for permanent disability [41]. In that case, the outcome was the disability pension, which is closely related to SA [42].

In contrast to these previous studies, we found no relationship between the SA course and 10 years of prior transitions in the labour market. A first alternative hypothesis from these findings could be that SA trajectories may be more related to a course that is nearer to labour market transitions (for instance, the previous 2 or 5 years). Furthermore, because a given health-related problem may interfere with an individual’s ability to meet current job demands, an imbalanced situation between these two conditions might be more likely to lead to accumulation of more days on SA compared to a prior adverse labour market transition pattern.

A second alternative hypothesis that could explain the results is that we did not measure employment quality during the prior working life. It is possible that studying a more comprehensive set of employment arrangements when defining the LMP patterns, including transitions among types of contract, working time, and occupations, could lead to a more specific LMP pattern that might relate to future SA behaviour. In this vein, one study has shown that working life patterns characterised by long-term exposure to blue-collar occupations, which are physically demanding and more subject to work accidents, have an effect on health outcomes that is similar to that of intermittent joblessness [14]. However, it should be considered that including a higher number of employment situations could increase the heterogeneity of patterns, which might attenuate the effect of an association between LMP patterns and health outcomes. Furthermore, as mentioned above, in some studies, the quality of employment was measured in terms of precarious employment using a multidimensional approach, yielding stronger associations with health outcomes than obtained by measuring one dimension [15]. However, most of those studies did not have a longitudinal perspective that could allow for consideration of the cumulative effects of employment quality over time [39].

Another hypothesis could be related to the impact of contextual variables, such as the economic crisis that began in 2008, Social Security coverage, and its relationship with the health system throughout the SA benefit system in the country. The impact of the economic crisis, which implied a general worsening of the labour market context, thus might have influenced the course of future SA, regardless of the individual’s employment pathway. Previous studies have focused on how contextual factors such as the unemployment rate or poor local economy affect SA. One such study showed a negative correlation between the unemployment rate and the probability of having long-term SA [8]. Another study showed that a poor local economy in terms of low municipal revenue and high unemployment rate was related to decreased self-certified SA [43]. In both cases, the threat of becoming unemployed because of a high level of unemployment discouraged workers from taking SA. However, the 3-year follow-up in our work could be too short to uncover lasting effects of the Great Recession on SA levels, especially when the most acute phase was in 2013, with an unemployment rate reaching 27% [44].

Finally, as several European studies have shown, differences in the probability of having SA exist according to characteristics of the sickness benefit system, such as the eligibility conditions and the level of generosity of SA compensation (i.e., wage replacement amount and benefit entitlement duration). For example, the probability of being on SA is higher in countries where employees are entitled to receive a full wage replacement in case of illness [45]. In contrast, another study showed a lag effect between levels of SA benefits on SA incidence, so that countries with relatively generous SA benefits show lower levels of SA in the long term. According to those authors, the generosity of SA benefits provided sufficient income support that helped beneficiaries overcome economic hardship and recover health [46]. Spain has specific conditions for access to SA (i.e., 180 days of paid contributions during the prior 5 years), relatively generous benefits (e.g., 60–75% wage replacement from the Social Security budget), and access to medical care provided by the National Health System. The cost of SA in Spain is shared by the employer (4th–15th day) and the Social Security system (16th onwards), unlike other countries where the employer pays the entire cost of SA [47]. However, because our study population was based exclusively on workers who have had SA, they were all entitled to full SA benefits, so our results may not be attributable to the eligibility conditions for access. In the present study, employees who did not accumulate days on SA were not included. Last but not least, a general practitioner grants medical certification from the National Health System, regardless of the Social Security system and companies. In this sense, a possible adverse effect of unstable employment on health may be buffered by the provision of universal health coverage by the National Health System.

Nevertheless, we found that men in their early working life stage who followed a pattern of increasing time in employment were less likely to increase their accumulation of future SA days. This result suggests that a stable transition into employment at the beginning of working life may have a protective effect on future health. However, according to several studies, young workers tend to enter into more insecure and precarious employment compared to older workers [48, 49], and temporary workers report higher job insecurity than those with permanent jobs and show lower SA rates [50]. In line with previous studies, we found that one-third of men at the early working life stage were employed in temporary jobs (Supplementary Table 4). An alternative explanation would be that low levels of SA are a reflection of an earlier return to work that can be stimulated by the loss of financial resources through receiving SA benefits instead of a full salary. This situation may particularly affect temporary workers because their jobs are often more precarious and employment conditions often worse, which could induce them to reduce SA length out of fear of job dismissal and being unable to fulfil their financial needs [50, 51].

One of the main limitations of this study is that workers without SA were excluded, which could have meant exclusion of those with better health status, those who had poor health status but were engaged in presenteeism, or those who did not qualify for SA benefits during the previous period, leading to selection bias. Also, we have classified workers according to their employment condition, medical diagnosis group, and the labour market state where they spent most of the time as a representative of the entire period, which could have led to a potential classification bias. A weekly labour state measure could have led to underestimation of short employment spells because approximately one-fifth of these were less than four days (results not shown). Another limitation comes from the use of two modelling methods based on simplifying assumptions, which may have implications for the robustness and reliability of the results; therefore, any conclusion derived should be considered carefully [17, 52]. In this regard, LMP patterns in the early working life cohorts may not represent homogeneous groups because their global ASW showed lower values than recommended. Likewise, the low stable SA trajectories showed the lowest values of assignment accuracy across WLCs. Nevertheless, the assessment of cluster analysis and latent classes showed adequate values for most of the groups. Also, the results are representative of salaried workers living in Catalonia and require caution with comparisons to other countries because differences in labour market regulations, social security systems (e.g., SA benefit generosity), and sociocultural context could shape SA in a distinct way [47]. Moreover, this study could not account for working conditions (only occupational category as a proxy) or health status before the SA course because the information was not available; therefore, a potential confounding bias might have affected our estimates. Notwithstanding, not controlling for initial health status is less an issue for those workers at early working life stages because bad health increases with age. The decreasing SA trajectories could represent not only workers who reduced accumulated SA days over time but also those who exited the labour market because of permanent disability or retirement, or who died during the 3-year follow-up. Nevertheless, in our study, workers in such situations represented only 4–8% of the population, depending on the trajectory group and working life stage. Furthermore, the administrative registers do not record a worker’s status when they are without contact with Social Security. Individuals could be unemployed without benefits and actively seeking a job, jobless and not seeking a job (i.e., outside the labour force or inactive), or in informal employment (i.e., working off the record, without a contract or social protection), and we do not know how these situations affect their health [53].

The study also has some strengths. First, we incorporated a life-course approach into the design. To our knowledge, few studies on occupational epidemiology have examined the relationship between 10 years of prior working life and the course of future health. Prior evidence on this issue has been based mostly on cross-sectional or longitudinal studies without a life-course approach [41, 54, 55]. In practice, the construction of patterns of LMP allows for consideration of the timing, order, and duration of given labour market states. Second, the study population comes from a Spanish workforce cohort (the WORKss cohort) [23], which has a large sample size and comprises high-quality registers. Third, employment history and SA data are register-based and medically certified, which allows for calculation of the exact starting and closing dates for each employment status period and SA episode. Fourth, the SA medical diagnosis certification is issued by a general practitioner from the National Health System, which implies higher validity of the diagnosis than self-reported SA measures [54].

## Conclusions

In conclusion, patterns of LMP during a 10-year prior working life are not related to future SA trajectories, regardless of the stage of the working life. Future studies should consider working life transitions closer to the SA course, with an employment quality approach framed with contextual factors, which may help in understanding of this relationship.

## Supplementary information

**Additional file 1: Supplementary Table 1.** (clustering quality average silhouette width).

**Additional file 2: Supplementary Table 2.** (model fit results for latent class growth analysis).

**Additional file 3: Supplementary Table 3.** (model adequacy assessment for latent class growth analysis).

**Additional file 4: Supplementary Table 4.** (distribution of salaried workers across labour market participation patterns by covariates).

## Data Availability

The datasets supporting the findings of this study are based on registers from the Spanish Social Security and the Catalan Institute for Medical and Health Evaluations. A record linkage agreement protocol between both institutions and the Centre for Research in Occupational Health ensures the confidentiality of the databases, which are anonymised to the authors and are not publicly available.

## Abbreviations

SA: sickness absence
LMP: labour market participation
WORKss cohort: The Spanish WORKing life Social Security cohort
ICD-10: International Classification of Diseases 10th version
WLC: working life cohort
LCGA: latent class growth analysis
