# Correction to: Sickness absence trajectories following labour market participation patterns: a cohort study in Catalonia (Spain), 2012–2014

**DOI:** 10.1186/s12889-020-09544-1

**Published:** 2020-10-01

**Authors:** Julio C. Hernando-Rodriguez, Laura Serra, Fernando G. Benavides, Monica Ubalde-Lopez

**Affiliations:** 1 Center for Research in Occupational Health (CiSAL), Pompeu Fabra University, Barcelona, Spain; 2 CIBER of Epidemiology and Public Health (CIBERESP), Madrid, Spain; 3 IMIM – Parc Salut Mar, Barcelona, Spain; 4 GRECS-Research Group on Statistics, Econometrics and Health, Faculty of Economics and Business, University of Girona (UdG), C/ Universitat de Girona, 10, 17071 Girona, Spain; 5 Barcelona Institute for Global Health (ISGlobal), Barcelona, Spain

**Correction to: BMC Public Health (2020) 20:1306**

**https://doi.org/10.1186/s12889-020-09396-9**

It was highlighted that in the original article [1] the legends of Figs. 1 and 2 were interchanged. This Correction article shows the Fig. 1 and Fig. 2 with their correct legends. The original article has been updated.

**Fig. 1:**
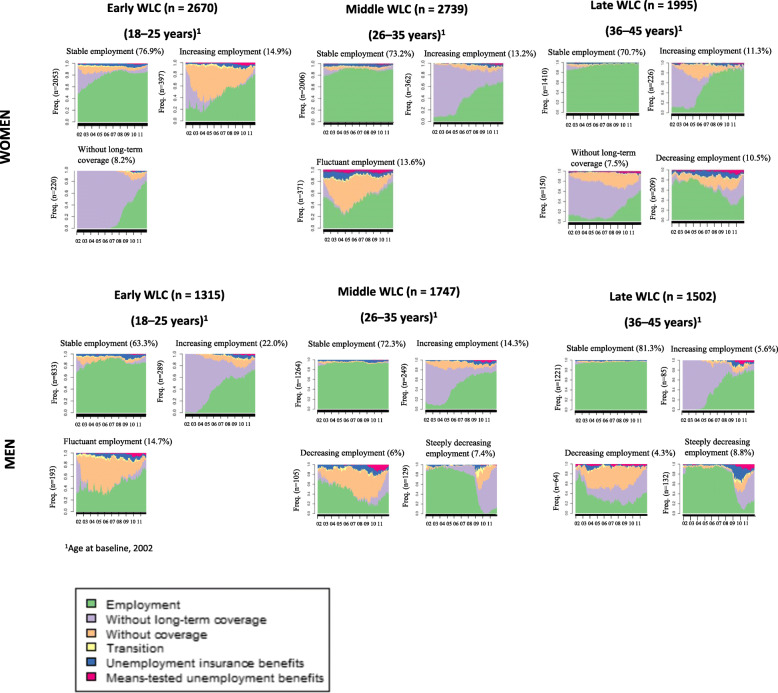
Labour market participation patterns in salaried workers with future sickness absence (> 15 accumulated days on sickness absence per quarter) across working life cohorts (WLCs) (*N* = 11,968). Catalonia, 2002–2011

**Fig. 2:**
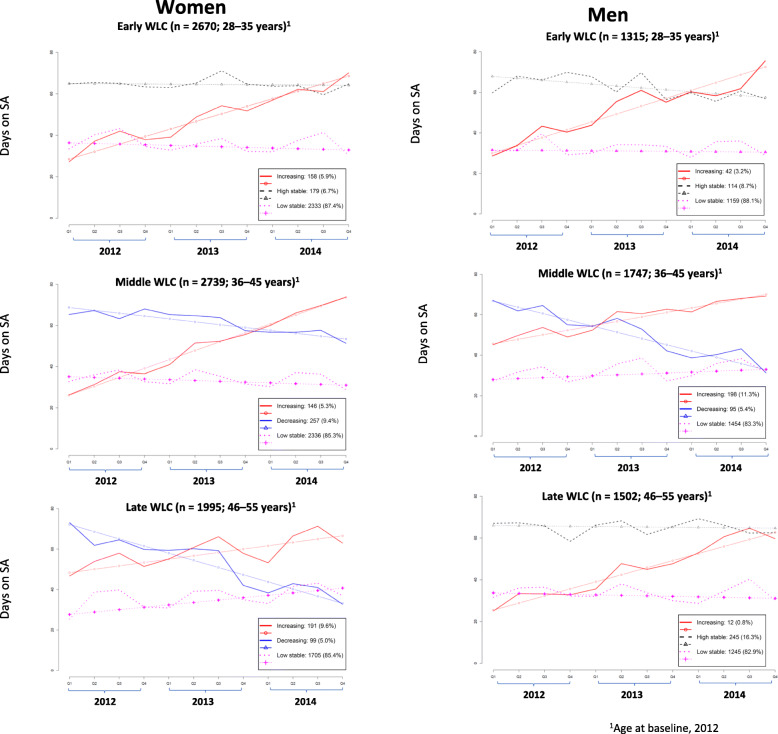
Sickness absence trajectories (> 15 accumulated days on sickness absence per quarter) in salaried workers across working life cohorts (WLCs) (N = 11,968). Catalonia, 2012–2014
