# Supplementary material for: Sickness absence trajectories following labour market participation patterns: a cohort study in Catalonia (Spain), 2012–2014
Source: BMC Public Health. 2020 Aug 27;20:1306. doi: 10.1186/s12889-020-09396-9 (PMC7453716; doi:10.1186/s12889-020-09396-9)
Supplement: Supplementary file 1 — Additional file 1: Supplementary Table 1. (clustering quality average silhouette width). [file 12889_2020_9396_MOESM1_ESM.docx]

| ASW ^a^ | Women | | |  | Men  **Supplementary Table 1.** Clustering quality by using the overall average silhouette width (ASW) for the entire 3-4 clusters and the ASW for each cluster by sex and working life cohort (WLC) groups. | | |
| --- | --- | --- | --- | --- | --- | --- | --- |
|  | Early WLC | Middle WLC | Late WLC |  | Early WLC | Middle WLC | Late WLC |
| Global | 0.35 | 0.47 | 0.52 |  | 0.39 | 0.51 | 0.63 |
| Cluster 1 | 0.39 | 0.61 | 0.73 |  | 0.53 | 0.66 | 0.74 |
| Cluster 2 | -0.03 | 0.26 | 0.13 |  | 0.26 | 0.10 | 0.35 |
| Cluster 3 | 0.67 | -0.07 | 0.17 |  | -0.01 | -0.04 | -0.03 |
| Cluster 4 | N/A | N/A | -0.17 |  | N/A | 0.29 | 0.04 |

^a^ Values close to 1: the sequence has been strongly well clustered; value 0: the sequence lies equally far away from any other cluster; value close to -1: the sequence lies on average much closer to another cluster than that assigned (misclassification).
