# Supplementary material for: Sickness absence trajectories following labour market participation patterns: a cohort study in Catalonia (Spain), 2012–2014
Source: BMC Public Health. 2020 Aug 27;20:1306. doi: 10.1186/s12889-020-09396-9 (PMC7453716; doi:10.1186/s12889-020-09396-9)
Supplement: Supplementary file 2 — Additional file 2: Supplementary Table 2. (model fit results for latent class growth analysis). [file 12889_2020_9396_MOESM2_ESM.docx]

**Supplementary Table 2.** Model fit results for latent class growth analysis of salaried women and men (>15 accumulated days on sickness absence per quarter) from early, middle, and late working life cohorts (WLCs) (N = 11,968). Catalonia, 2012–2014.

^a^ Bayesian information criterion; ^b^ Lo-Mendell-Rubin adjusted likelihood ratio test; ^c^ bootstrapped likelihood ratio test; ^d^ sample size per class based on most likely membership

|  |  | No. Of classes | BIC ^a^ | Sample size–adjusted BIC ^b^ | LMR-LRT ^c^ | BLRT ^d^ | Entropy | Sample size per class ^e^ |
| --- | --- | --- | --- | --- | --- | --- | --- | --- |
| Women | Early WLC | 2 | 36490 | 36436 | 0.002 | <0.001 | 0.62 | 2398/272 |
|  |  | 3 | 36478 | 36414 | 0.008 | <0.001 | 0.52 | 2333/179/158 |
|  |  | 4 | 36481 | 36408 | 0.106 | <0.001 | 0.45 | 94/84/2257/235 |
|  | Middle WLC | 2 | 38429 | 38375 | <0.001 | <0.001 | 0.65 | 338/2401 |
|  |  | 3 | 38400 | 38337 | <0.001 | <0.001 | 0.55 | 2336/257/146 |
|  |  | 4 | 38424 | 38351 | 0.497 | 1.000 | 0.57 | 160/0/2395/184 |
|  | Late WLC | 2 | 30457 | 30403 | 0.048 | <0.001 | 0.54 | 1732/263 |
|  |  | 3 | 30450 | 30386 | 0.432 | <0.001 | 0.51 | 1705/99/191 |
|  |  | 4 | 30463 | 30390 | 0.067 | 0.065 | 0.43 | 113/127/1684/71 |
| Men | Early WLC | 2 | 16680 | 16626 | 0.047 | <0.001 | 0.68 | 175/1140 |
|  |  | 3 | 16681 | 16618 | 0.272 | <0.001 | 0.64 | 1159/114/42 |
|  |  | 4 | 16702 | 16629 | 0.746 | 1.000 | 0.69 | 1160/36/0/119 |
|  | Middle WLC | 2 | 23605 | 23551 | 0.008 | <0.001 | 0.63 | 268/1479 |
|  |  | 3 | 23597 | 23534 | 0.137 | <0.001 | 0.53 | 1454/95/198 |
|  |  | 4 | 23598 | 23525 | 0.020 | <0.001 | 0.47 | 137/76/1420/114 |
|  | Late WLC | 2 | 20796 | 20742 | <0.001 | <0.001 | 0.67 | 241/1261 |
|  |  | 3 | 20807 | 20744 | 0.400 | 0.013 | 0.60 | 1245/245/12 |
|  |  | 4 | 20829 | 20756 | 0.516 | 1.000 | 0.66 | 1247/10/0/245 |
