# Supplementary material for: Sickness absence trajectories following labour market participation patterns: a cohort study in Catalonia (Spain), 2012–2014
Source: BMC Public Health. 2020 Aug 27;20:1306. doi: 10.1186/s12889-020-09396-9 (PMC7453716; doi:10.1186/s12889-020-09396-9)
Supplement: Supplementary file 3 — Additional file 3: Supplementary Table 3. (model adequacy assessment for latent class growth analysis). [file 12889_2020_9396_MOESM3_ESM.docx]

**Supplementary Table 3.** Model adequacy assessment for latent class growth analysis of salaried women and men (>15 accumulated days on sickness absence per quarter) from early, middle, and late working life cohorts (WLCs) (N = 11,968). Catalonia, 2012–2014.

|  |  |  | Model estimate (πj) ^a^ | P_j_ (proportion classified in group j) ^b^ | Ave. PP ^c^ | OCCj ^d^ | \|π_j_-Pj\|^e^ |
| --- | --- | --- | --- | --- | --- | --- | --- |
| Women | Early WLC | Low stable | 0.73 | 0.87 | 0.820 | 1.66 | 0.14 |
|  |  | High stable | 0.11 | 0.07 | 0.684 | 17.14 | 0.05 |
|  |  | Increasing | 0.16 | 0.06 | 0.560 | 6.92 | 0.10 |
|  | Middle WLC | Low stable | 0.74 | 0.85 | 0.840 | 1.85 | 0.11 |
|  |  | Decreasing | 0.14 | 0.09 | 0.685 | 13.88 | 0.04 |
|  |  | Increasing | 0.13 | 0.05 | 0.637 | 12.26 | 0.07 |
|  | Late WLC | Low stable | 0.72 | 0.85 | 0.818 | 1.72 | 0.13 |
|  |  | Decreasing | 0.12 | 0.05 | 0.703 | 16.61 | 0.08 |
|  |  | Increasing | 0.15 | 0.10 | 0.633 | 9.60 | 0.06 |
| Men | Early WLC | Low stable | 0.79 | 0.88 | 0.881 | 1.97 | 0.09 |
|  |  | High stable | 0.11 | 0.09 | 0.696 | 17.65 | 0.03 |
|  |  | Increasing | 0.10 | 0.03 | 0.652 | 17.84 | 0.06 |
|  | Middle WLC | Low stable | 0.71 | 0.83 | 0.827 | 1.96 | 0.12 |
|  |  | Decreasing | 0.14 | 0.05 | 0.720 | 16.08 | 0.08 |
|  |  | Increasing | 0.15 | 0.11 | 0.687 | 12.17 | 0.04 |
|  | Late WLC | Low stable | 0.74 | 0.83 | 0.862 | 2.19 | 0.09 |
|  |  | High stable | 0.17 | 0.16 | 0.758 | 14.81 | 0.01 |
|  |  | Increasing | 0.09 | 0.01 | 0.642 | 19.24 | 0.08 |

^a^ Posterior probability of group membership; ^b^ Pj, proportion of the sample assigned based on maximum posterior probability assignment rule; ^c^ average posterior probability of assignment; ^d^ the odds of correct classification (OCC) = the ratio between the OCC into group j based on the maximum probability classification rule (AvePP) and the OCC into group j based on random assignment using the model-estimated proportion (πj); ^e^ difference between the estimated group probabilities and the proportion of the sample assigned to the group (i.e., mismatch).
