# Supplementary material for: Sickness absence trajectories following labour market participation patterns: a cohort study in Catalonia (Spain), 2012–2014
Source: BMC Public Health. 2020 Aug 27;20:1306. doi: 10.1186/s12889-020-09396-9 (PMC7453716; doi:10.1186/s12889-020-09396-9)
Supplement: Supplementary file 4 — Additional file 4: Supplementary Table 4. (distribution of salaried workers across labour market participation patterns by covariates). [file 12889_2020_9396_MOESM4_ESM.docx]

| **Supplementary Table 4.** Distribution of salaried women and men from early, middle, and late working life cohorts (WLC) (N = 11,968) across labour market participation patterns (>15 accumulated days on sickness absence per quarter) by type of contract, working time, occupational category, and income. Catalonia, 2002­–2011. | | | | | | | | | | | | | | | | |
| --- | --- | --- | --- | --- | --- | --- | --- | --- | --- | --- | --- | --- | --- | --- | --- | --- |
|  | **Early WLC (N = 2670)** | | | |  | **Middle WLC (N = 2739)** | | | | |  | **Late WLC (N = 1995)** | | | | |
| **Women** | **Stable employment (76.9%)** | **Increasing employment (14.9%)** | **Without long-term coverage (8.2%)** |  |  | **Stable employment (73.2%)** | **Increasing employment (13.2%)** | **Fluctuant employment (13.6%)** |  |  |  | **Stable employment (70.7%)** | **Increasing employment (11.3%)** | **Without long-term coverage (7.5%)** | **Decreasing employment (10.5%)** |  |
|  | N (%) | N (%) | N (%) | *p*^a^ |  | N (%) | N (%) | N (%) | *p*^a^ |  |  | N (%) | N (%) | N (%) | N (%) | *p*^a^ |
| **Type of contract** |  |  |  |  |  |  |  |  |  |  |  |  |  |  |  |  |
| Permanent contract | 1612 (78.8) | 221 (55.7) | 93 (51.4) | <.001 |  | 1711 (85.6) | 217 (65.0) | 231 (62.6) | <.001 |  |  | 1262 (90.3) | 164 (74.9) | 69 (52.3) | 153 (74.3) | <.001 |
| Temporary contract | 435 (21.3) | 176 (44.3) | 88 (48.6) |  |  | 289 (14.5) | 117 (35.0) | 138 (37.4) |  |  |  | 135 (9.7) | 55 (25.1) | 63 (47.7) | 53 (25.7) |  |
| **Working time** |  |  |  |  |  |  |  |  |  |  |  |  |  |  |  |  |
| Full-time | 1752 (85.3) | 287 (72.3) | 171 (77.7) | <.001 |  | 1669 (83.2) | 261 (72.3) | 252 (67.9) | <.001 |  |  | 1187 (84.2) | 152 (67.3) | 81 (54.4) | 158 (75.6) | <.001 |
| Part-time: >50% up to 99% | 217 (10.6) | 54 (13.6) | 23 (10.5) |  |  | 250 (12.5) | 55 (15.2) | 61 (16.4) |  |  |  | 147 (10.4) | 33 (14.6) | 28 (18.8) | 20 (9.6) |  |
| ≤50% | 84 (4.1) | 56 (14.1) | 26 (11.8) |  |  | 87 (4.3) | 45 (12.5) | 58 (15.6) |  |  |  | 76 (5.4) | 41 (18.1) | 40 (26.9) | 31 (14.8) |  |
| **Occupational category** |  |  |  |  |  |  |  |  |  |  |  |  |  |  |  |  |
| Skilled non-manual | 402 (19.6) | 110 (27.7) | 36 (19.8) | <.001 |  | 473 (23.6) | 48 (14.3) | 55 (14.9) | <.001 |  |  | 278 (19.8) | 16 (7.3) | 10 (7.6) | 21 (10.1) | <.001 |
| Skilled manual | 305 (14.9) | 37 (9.3) | 46 (25.3) |  |  | 267 (13.3) | 61 (18.2) | 65 (17.6) |  |  |  | 218 (15.6) | 43 (19.6) | 29 (22.0) | 60 (29.0) |  |
| Unskilled non-manual | 1226 (59.8) | 214 (53.9) | 57 (31.3) |  |  | 1102 (55.0) | 139 (41.5) | 174 (47.0) |  |  |  | 706 (50.4) | 89 (40.6) | 38 (28.8) | 84 (40.6) |  |
| Unskilled manual | 116 (5.7) | 36 (9.1) | 43 (23.6) |  |  | 160 (8.0) | 87 (26.0) | 76 (20.5) |  |  |  | 199 (14.2) | 71 (32.4) | 55 (41.7) | 42 (20.3) |  |
| **Income in quartiles** |  |  |  |  |  |  |  |  |  |  |  |  |  |  |  |  |
| High | 758 (36.9) | 106 (26.7) | 40 (18.4) | <.001 |  | 721 (35.9) | 57 (15.8) | 48 (12.9) | <.001 |  |  | 475 (33.7) | 16 (7.1) | 15 (10.0) | 36 (17.2) | <.001 |
| Middle-high | 648 (31.6) | 94 (23.7) | 44 (20.2) |  |  | 619 (30.9) | 80 (22.1) | 79 (21.3) |  |  |  | 482 (34.2) | 48 (21.2) | 17 (11.3) | 48 (23.0) |  |
| Middle-low | 453 (22.1) | 91 (22.9) | 45 (20.6) |  |  | 442 (22.0) | 113 (31.2) | 106 (28.6) |  |  |  | 323 (22.9) | 85 (37.6) | 37 (24.7) | 64 (30.6) |  |
| Low | 194 (9.5) | 106 (26.7) | 89 (40.8) |  |  | 224 (11.2) | 112 (30.9) | 138 (37.2) |  |  |  | 130 (9.2) | 77 (34.1) | 81 (54.0) | 61 (29.2) |  |
| Total | 2,053 (100.0) | 397 (100.0) | 220 (100.0) |  |  | 2,006 (100.0) | 362 (100.0) | 371 (100.0) |  |  |  | 1,410 (100.0) | 226 (100.0) | 150 (100.0) | 209 (101.0) |  |
|  | **Early WLC (N = 1315)** | | | |  | **Middle WLC (N = 1747)** | | | | |  | **Late WLC (N = 1502)** | | | | |
| **Men** | **Stable employment (63.3%)** | **Increasing employment (22.0%)** | **Fluctuant employment (14.7%)** |  |  | **Stable employment (72.3%)** | **Increasing employment (14.3%)** | **Decreasing employment (6.0%)** | **Steep labour market exit (7.4%)** |  |  | **Stable employment (81.3%)** | **Increasing employment (5.6%)** | **Decreasing employment (4.3%)** | **Steep entry into unemployment (8.8%)** |  |
|  | N (%) | N (%) | N (%) | *p*^a^ |  | N (%) | N (%) | N (%) | N (%) | *p*^a^ |  | N (%) | N (%) | N (%) | N (%) | *p*^a^ |
| **Type of contract** |  |  |  |  |  |  |  |  |  |  |  |  |  |  |  |  |
| Permanent contract | 693 (83.7) | 189 (66.6) | 95 (49.5) | <.001 |  | 1115 (88.7) | 161 (66.0) | 53 (51.0) | 113 (89.7) | <.001 |  | 1122 (92.4) | 55 (68.8) | 43 (68.3) | 109 (82.6) | <.001 |
| Temporary contract | 135 (16.3) | 95 (33.5) | 97 (50.5) |  |  | 142 (11.3) | 83 (34.0) | 51 (49.0) | 13 (10.3) |  |  | 92 (7.6) | 25 (31.3) | 20 (31.8) | 23 (17.4) |  |
| **Working time** |  |  |  |  |  |  |  |  |  |  |  |  |  |  |  |  |
| Full-time | 809 (97.1) | 253 (87.5) | 163 (84.5) | <.001 |  | 1222 (96.7) | 221 (89.1) | 95 (91.4) | 120 (93.0) | <.001^b^ |  | 1190 (97.5) | 72 (86.8) | 49 (76.6) | 128 (97.0) | <.001^b^ |
| Part-time: >50% up to 99% | 13 (1.6) | 14 (4.8) | 16 (8.3) |  |  | 22 (1.7) | 14 (5.7) | 5 (4.8) | 4 (3.1) |  |  | 16 (1.3) | 7 (8.4) | 1 (1.6) | 1 (0.8) |  |
| ≤50% | 11 (1.3) | 22 (7.6) | 14 (7.3) |  |  | 20 (1.6) | 13 (5.2) | 4 (3.9) | 5 (3.9) |  |  | 15 (1.2) | 4 (4.8) | 14 (21.9) | 3 (2.3) |  |
| **Occupational category** |  |  |  |  |  |  |  |  |  |  |  |  |  |  |  |  |
| Skilled non-manual | 54 (6.5) | 38 (13.3) | 29 (15.1) | <.001 |  | 202 (16.0) | 30 (12.2) | 13 (12.5) | 10 (7.9) | <.001 |  | 263 (21.6) | 9 (11.0) | 6 (9.5) | 12 (9.1) | <.001 |
| Skilled manual | 444 (53.5) | 99 (34.6) | 54 (28.1) |  |  | 541 (43.0) | 96 (38.9) | 49 (47.1) | 81 (64.3) |  |  | 481 (39.5) | 35 (42.7) | 33 (52.4) | 82 (62.1) |  |
| Unskilled non-manual | 217 (26.1) | 77 (26.9) | 73 (38.0) |  |  | 401 (31.9) | 50 (20.2) | 28 (26.9) | 22 (17.5) |  |  | 374 (30.7) | 18 (22.0) | 19 (30.2) | 28 (21.2) |  |
| Unskilled manual | 115 (13.9) | 72 (25.2) | 36 (18.8) |  |  | 115 (9.1) | 71 (28.7) | 14 (13.5) | 13 (10.3) |  |  | 101 (8.3) | 20 (24.4) | 5 (7.9) | 10 (7.6) |  |
| **Income in quartiles** |  |  |  |  |  |  |  |  |  |  |  |  |  |  |  |  |
| High | 275 (33.0) | 53 (18.3) | 35 (18.1) | <.001 |  | 393 (31.1) | 30 (12.1) | 23 (21.9) | 45 (34.9) | <.001 |  | 348 (28.5) | 12 (14.1) | 17 (26.6) | 26 (19.7) | <.001 |
| Middle-high | 263 (31.6) | 81 (28.0) | 40 (20.7) |  |  | 409 (32.4) | 42 (16.9) | 15 (14.3) | 58 (45.0) |  |  | 397 (32.5) | 10 (11.8) | 11 (17.2) | 51 (38.6) |  |
| Middle-low | 198 (23.8) | 81 (28.0) | 49 (25.4) |  |  | 292 (23.1) | 80 (32.1) | 24 (22.9) | 12 (9.3) |  |  | 303 (24.8) | 25 (29.4) | 12 (18.8) | 31 (23.5) |  |
| Low | 97 (11.6) | 74 (25.6) | 69 (35.8) |  |  | 170 (13.5) | 97 (39.0) | 43 (41.0) | 14 (10.9) |  |  | 173 (14.2) | 38 (44.7) | 24 (37.5) | 24 (18.2) |  |
| Total | 833 (100.0) | 289 (100.0) | 193 (100.0) |  |  | 1264 (100.0) | 249 (100.0) | 105 (100.0) | 129 (101.0) |  |  | 1221 (100.0) | 85 (100.0) | 64 (100.0) | 132 (101.0) |  |

Missing values in type of contract (TC), working time (WT), occupational category (OC), and income (I): persons N (%) in the early cohort (women, stable employment—TC: 6 (0.29); OC: 4 (0.19); delayed employment—TC: 39 (17.73); OC: 38 (17.27); I—2 (0.91); and men: stable employment—TC: 5 (0.60); OC: 3 (0.36); increasing employment—TC: 5 (1.73); OC: 3 (1.04); fluctuant employment—TC: 1 (0.52); OC: 1 (0.52)); middle cohort (women, stable employment—TC: 6 (0.30); OC: 4 (0.20); increasing employment—TC: 28 (7.73); WT: 1 (0.28); OC: 27 (7.46); fluctuant employment—TC: 2 (0.54); OC: 1 (0.27); and men, stable employment—TC: 7 (0.55); OC: 5 (0.40); increasing employment—TC: 5 (2.01); WT: 1 (0.40); OC: 2 (0.80); decreasing employment—TC: 1 (0.95); WT: 1 (0.95); OC: 1 (0.95); steeply decreasing employment—TC: 3 (2.33); OC: 3 (2.33)); and late cohort (women, stable employment—TC: 13 (0.92); OC: 9 (0.64); increasing employment—TC: 7 (3.10); OC: 7 (3.10); delayed employment—TC: 18 (12.00); WT: 1 (0.67); OC: 18 (12.00); decreasing employment—TC: 3 (1.44); OC: 2 (0.96); and men, stable employment—TC: 7 (0.57); OC: 2 (0.16); increasing employment—TC: 5 (5.88); WT: 2 (2.35); OC: 3 (3.53); decreasing employment—TC: 1 (1.56); OC: 1 (1.56)). Income in quartiles based on the average monthly income in the early cohort (women—high: 4,123€; middle-high: 1,512€; middle-low: 1,141€; low: 865€; and men—high: 4,252€; middle-high: 1,695€; middle-low: 1,338€; low: 1,063€), middle cohort (women—high: 4,746€; middle-high: 1,781€; middle-low: 1,234€; low: 885€; and men—high: 5,257€; middle-high: 2,230€; middle-low: 1,603€; low: 1,224€), and late cohort (women—high: 5,262€; middle-high: 1,855€; middle-low: 1,197€; low: 822€; and men—high: 5,313€; middle-high: 2,629€; middle-low: 1,786€; low: 1,311€). ^a^Chi-squared tests. ^b^Fisher’s exact tests.
